# Supplementary material for: Correlation of breast cancer microcirculation construction with tumor stem cells (CSCs) and epithelial-mesenchymal transition (EMT) based on contrast-enhanced ultrasound (CEUS)
Source: PLoS One. 2021 Dec 21;16(12):e0261138. doi: 10.1371/journal.pone.0261138 (PMC8691655; doi:10.1371/journal.pone.0261138)
Supplement: Attachment — Submitted filename: 2021 04 30 Response letter revised.docx [file pone.0261138.s002.docx]

Dear editor,

On behalf of all co-authors, I would like to thank you for your letter concerning our manuscript. We greatly appreciate you and the reviewers for the critical reading of our manuscript and giving us the favorable comments and instructive suggestions.

We have carefully proof-read and revised the manuscript according to the reviewers’ comments and the editorial notes. Here we submit the revised manuscript. In the following pages, the responses to the comments are described point-by-point.

Once again, we want to extend our appreciation to you and the reviewers for the valuable and helpful comments. We sincerely hope that the manuscript has been revised to your satisfaction, and we would be grateful if the manuscript could be considered for publication.

In addition, we would like to update our Funding Statement as follows:

This work was supported by Natural Science Foundation of Xinjiang Uygur Autonomous Region (2018D01C276).

Sincerely yours,

Jianbing Ding

Basic Medical College, Xinjiang Medical University, No. 393 Xinyi Road, Urumqi 830011, Xinjiang, China. Tel: 86-18999230065. Email: dingjb1234@ aliyun.com & 1601379937@qq.com

**The reviewer’s comments and the authors’ responses:**

Please use the space provided to explain your answers to the questions above. You may also include additional comments for the author, including concerns about dual publication, research ethics, or publication ethics. (Please upload your review as an attachment if it exceeds 20,000 characters)

**Reviewer #1:** The authors have demonstrated that the correlation between the parameters of less invasive CEUS and the expression level of CSC/EMT markers in breast cancer by IHC and RT-PCR. However, the experimental results are not sufficient to state their conclusions.

Response: To validate our conclusions, we have further performed Western blot analysis of CSC-related and EMT-related markers in tumor and tumor adjacent tissues. The results showed that compared with normal tissues adjacent to cancer, the breast cancer tissues had significantly up-regulated protein expression levels of CD44, N-cadherin, and β-catenin, but significantly down-regulated protein expression levels of E-cadherin and CD24 (Fig. 3A and 3B). No significant difference was observed in the Vimentin protein expression levels between the cancer and adjacent tissues. We have added this result to the revised Fig. 3 and modified the revised manuscript accordingly.

However, further studies are still needed to validate our results.

The authors must evaluate IHC either in the same cell (double staining) or by the staining using serial sections.

Response: In immunohistochemistry, the staining color position and depth indicated the expression levels and patterns, and it was difficult to perform double staining on the very one section. Therefore, these indicators were subjected to IHC staining on serial sections.

However, the reviewer raised a very good point. We will do as suggested in future studies.

RT-PCR should use RNA extracted from cancer cells or normal cells recovered by laser microdissection, rather than RNA extracted from whole tissues.

Response: The tumor samples were obtained under the guidance of B-ultrasound. The hyperplastic gland tissues were used as control. Due to limited funding, we did not recover the cells by laser microdissection. However, the reviewer raised a very good point. We will do as suggested in future studies.

Furthermore, it is necessary to use the TaqMan probe method, which has a high quantitative yield, instead of the cybr green method, which is a semi-quantitative method.

Response: The quantitative real-time PCR was performed to detect the mRNA expression levels of the target genes. However, the reviewer raised a very good point. We will do as suggested in future studies.

Minor points:

1) Please insert scale bars in Fig.1.

Response: In fact, the figures were taken at the magnification of 200×, which has been added in the figure legends.

2) The information of antibodies of IHC should be written down in Materials and Methods.

Response: According to the comment, we have added the information of IHC antibodies in Materials and Methods section of the revised manuscript.

3) Please describe the detailed statistical analysis methods in Materials and Methods.

Response: If the data conformed to the normal distribution, the single-factor analysis of variance was performed. Multiple comparisons were performed with the sidak method for the data with homogeneity of variance, while the Tamhane method was used for the data with the heterogeneity of variance. The data without normal distribution were first subjected to the logarithmical transform for normalization, and then the above-mentioned one-way analysis of variance method was performed for statistical analysis, or the Wilcoxon rank sum test was used. Correlation analysis of the expression index was performed with the Pearson correlation.

4) There are inappropriate citations, such as quoting a lung cancer paper even though it is a breast cancer citation. Please review the selection of cited papers.

Response: Sorry for the inappropriate citations. According to the comment, corresponding changes have been made herein.

5) There are many misspellings. Please check the spelling.

Response: According to the comment, we have further gotten editorial help to improve the English writing of the revised manuscript. Please check!

**Reviewer #2:** Contrast-enhanced ultrasound (CEUS) procedure carried out with new medical equipment and new-generation micro-bubble contrast reagents has been suggested to increase the accuracy in providing information on the microcirculation. The overall aim of the research described in this manuscript is to assess whether CEUS-based features may provide prognostic type of information. In particular, CEUS-based microcirculation features are examined for correlation with cancer-stem cell (CSC)-like subpopulations including those undergoing epithelial-mesenchymal transition (EMT). The rationale for focusing on tumor cells with CSC and EMT-like characteristics relates to potential roles in tumor heterogeneity and aggressiveness. The manuscript including the background, methods, and results would benefit from better language use and inclusion of information in terms of providing a clearer understanding of the rationale and the objectives of the study, the methodologies used, data interpretation including implications.

Specific Comments-

1. Some of the information presented in the discussion should be considered to be moved to the background to help provide a basis for the study.

Response: According to the comment, corresponding changes have been made herein.

2. Several published studies (eg Ji et al, 2017. Clin Hemorheol Microcirc, Zhao et al, 2017 Onco Targets Ther, Cao et al, 2014. Ultrasound Med Biol, Wang et al, 2017, PLoS One, and Vraka et al, 2918 In Vivo) have assessed the correlation between CEUS-based parameters and prognostic markers in breast cancer should be alluded to and referenced in the background and/or discussion.

Response: According to the comment, we have added the following reference to the Discussion section. Please check!

Reference:

Vraka I, Panourgias E, Sifakis E, Koureas A, Galanis P, Dellaportas D, Gouliamos A, Antoniou A. In Vivo.Correlation Between Contrast-enhanced Ultrasound Characteristics (Qualitative and Quantitative) and Pathological Prognostic Factors in Breast Cancer. 2018;32(4):945-954. Epub 2018/07/30. doi: 10.21873/invivo.11333. PubMed PMID: 29936484;PubMed Central PMCID: PMC6117754.

3. The reason for use of 2D US prior to the CEUS should be included in the method.

Response: In fact, for the CEUS, the contrast mode should be conducted on the basis of a clear two-dimensional ultrasound image. Although the results needed not to be analyzed for the two-dimensional ultrasound, the operation method would be necessary, which represented the diagnostic rule for contrast ultrasound. However, these methodological information needed not to be explained in the study.

4. A figure that depicts the various parameters obtained related to the CEUS describe on page 6 in the methods should be included (see ). The functional implications of these parameters in terms of circulation/perfusion should be clearly stated to help understand their significance when described in the result sections. Use of full names (and not abbreviations) is recommended throughout the manuscript.

Response: According to the comment, corresponding information has been added herein. For example, we have used the full names instead of abbreviations according to the suggestion.

5. A rationale should be provided for the criteria used to follow the various biomarkers described in the IHC section of the method. The nature of the antibodies used what they recognize on target proteins should be included. What is the rationale for looking for cytoplasmic localization of CD24? β-catenin localization in cytoplasmic/nucleus can relate to WNT-pathway activation and potentially EMT, given this was this aspect inspected? How epithelial/carcinoma vs stromal cells distinguished? Where antibodies for cytokeratins used?

Response: According to the comment, corresponding information concerning the primary antibodies and the criteria for expression evaluation have been added in the revised manuscript.

Moreover, based on the Human-Protein-Atlas database, CD24 is located in the cytoplasm. The activation of the WNT pathway would cause the translocation of β-catenin from the cytoplasm to the nucleus [1]. In the nucleus, β-catenin combines with the LEF/TCF transcription factor to replace the auxiliary inhibitor, and this combination recruits the additional auxiliary activators of the Wnt target genes to promote the process of EMT [2]. There have been articles [3] reporting that CD24 is the target of Wnt in colorectal cancers. Therefore, from database predictions and previous literature, the WNT pathway activation and potential EMT would affect the CD24 cytoplasmic localization.

In addition, through information retrieving, the keratin (epithelial cells) and vimentin (stromal cells) are used to distinguish the epithelial cells from the stromal cells.

References:

[1] Borgal L, Habbig S, Hatzold J, Liebau MC, Dafinger C, Sacarea I, Hammerschmidt M, Benzing T, Schermer B. The ciliary protein nephrocystin-4 translocates the canonical Wnt regulator Jade-1 to the nucleus to negatively regulate β-catenin signaling. J Biol Chem. 2012 Jul 20;287(30):25370-80.

[2] Kobayashi W, Ozawa M. The epithelial-mesenchymal transition induced by transcription factor LEF-1 is independent of β-catenin. Biochem Biophys Rep. 2018 Jun 12;15:13-18.

[3] Chen YC, Lee TH, Tzeng SL. Reduced DAXX Expression Is Associated with Reduced CD24 Expression in Colorectal Cancer. Cells. 2019 Oct 12;8(10):1242.

6. EMT induction requires the expression of transcription factors. Thus, to better show the relationship of the biomarkers used, it will be important to assess the expression (correlation) of some of EMT-TF with the biomarkers and the CEUS-features tested in the current study.

Response: Based on the PROMO database, Genecards database and UCSC database, the transcription factors related to EMT indicators were predicted. The common transcription factors included the SP1, YY-1, etc. Through literature searching, the transcription factors were all related to EMT, which regulated the process of EMT through modulating the gene transcription. It has been reported that the prepared nanobubbles showed high affinity and specificity to breast cancer cells and tumors with the neuropeptide YY 1 receptors, with minimal toxicity and damages to the organs [1]. Biodegradable photoluminescent nanobubbles were used as ultrasound contrast agents for targeted breast cancer imaging. The transcription factor prediction results were obtained as attached files.

Reference:

[1] Li J, Tian Y, Shan D, Gong A, Zeng L, Ren W, Xiang L, Gerhard E, Zhao J, Yang J, Wu A. Neuropeptide Y Y1 receptor-mediated biodegradable photoluminescent nanobubbles as ultrasound contrast agents for targeted breast cancer imaging. Biomaterials. 2017 Feb;116:106-117.
