# Supplementary material for: Correlation of breast cancer microcirculation construction with tumor stem cells (CSCs) and epithelial-mesenchymal transition (EMT) based on contrast-enhanced ultrasound (CEUS)
Source: PLoS One. 2021 Dec 21;16(12):e0261138. doi: 10.1371/journal.pone.0261138 (PMC8691655; doi:10.1371/journal.pone.0261138)
Supplement: Attachment — Submitted filename: 2021 08 25 Response letter revised.docx.docx [file pone.0261138.s003.docx]

Dear editor,

We are resubmitting the Manuscript ID PONE-D-20-18825R1 entitled Correlation of breast cancer microcirculation construction with tumor stem cells (CSCs) and epithelial-mesenchymal transition (EMT) based on contrast-enhanced ultrasound (CEUS) to PLOS ONE. Our responses to the comments by the reviewers are outlined below. Please also see the revised manuscript for details. For easily reading, we use the TRACK function of MICROSOFT WORD. Please simply select “Accept changes” to get rid of the TRACK markers if you do not like the tracks.

In addition, we would like to update our Funding Statement as follows:

This work was supported by the Supporting Project from Xinjiang Science and Technology Department (No.2020E0269).

**Additional Editor Comments:**

As evident from the Reviewer's comments, it is critical to re-address the images in Figure 1. It is very hard to see how the images provided come from serial sections, or how they correlate with each other, so that one can extract meaningful conclusions. Please provide a revised Figure 1 with appropriate images, and/or low magnifications of the images to allow for evaluation of location of panels and potential co-localizations, as well as the assessment of the same tumor.

Response: As suggested, we have further provided a revised Figure 2 (the original Figure 1) with high and low magnifications of the same tumor or normal sites. We have also made corresponding changes in the revised manuscript. Please check!

**Reviewers' comments:**

Reviewer #1: They have not essentially revised for my major concerns.

My biggest concerns are below. Although authors described that they use the serial section for IHC, but as far as I can see in the picture in Figure 1, I can not think that they have observed the same tumor or normal site in the continuous section. The photos should be shown the same tumor or normal site in the serial section.

Response: As suggested, we have further provided a revised Figure 2 (the original Figure 1) with high and low magnifications of the same tumor or normal sites. We have also made corresponding changes in the revised manuscript. Please check!

In figure4, the amounts of N-cadherin, Beta-catenin, and Vimentin proteins in these representative photos are not consistent with these quantitative graphs.

Response: As suggested, we have modified the representative and quantitative Western blot results in the revised Figure 4 to ensure that the amounts of N-cadherin, Beta-catenin, and Vimentin proteins in these representative photos were consistent with these quantitative graphs. We have also made corresponding changes in the revised manuscript. Please check!

Thus, I think that their experimental results are inadequate to draw their conclusions.

Response: As suggested, we have modified some results and some conclusion to ensure that the conclusions were supported by the experimental results. For example, the overstated conclusions have been modified. Please check the revised manuscript for details.
