# Supplementary material for: Correlation of breast cancer microcirculation construction with tumor stem cells (CSCs) and epithelial-mesenchymal transition (EMT) based on contrast-enhanced ultrasound (CEUS)
Source: PLoS One. 2021 Dec 21;16(12):e0261138. doi: 10.1371/journal.pone.0261138 (PMC8691655; doi:10.1371/journal.pone.0261138)
Supplement: Attachment — Submitted filename: 2021 11 19 response to reviewer letter revised.docx [file pone.0261138.s004.docx]

Dear editor,

We are resubmitting the Manuscript ID PONE-D-20-18825R2 entitled Correlation of breast cancer microcirculation construction with tumor stem cells (CSCs) and epithelial-mesenchymal transition (EMT) based on contrast-enhanced ultrasound (CEUS) to PLOS ONE. Our responses to the comments by the reviewers are outlined below. Please also see the revised manuscript for details. For easily reading, we use the TRACK function of MICROSOFT WORD. Please simply select “Accept changes” to get rid of the TRACK markers if you do not like the tracks.

In addition, we would like to update our Funding Statement as follows:

This work was supported by the project of scientific and technological assistance to Xinjiang (No.2020E0269).

**Journal Requirements:**

Please review your reference list to ensure that it is complete and correct. If you have cited papers that have been retracted, please include the rationale for doing so in the manuscript text, or remove these references and replace them with relevant current references. Any changes to the reference list should be mentioned in the rebuttal letter that accompanies your revised manuscript. If you need to cite a retracted article, indicate the article’s retracted status in the References list and also include a citation and full reference for the retraction notice.

Response: We confirm that the reference list is complete and correct. No further changes to the reference list are needed. We did not cite any retracted papers.

**Reviewers' comments:**

**Reviewer's Responses to Questions**

**Reviewer #1:** I think it has improved. The IHC analysis result, which was a major concern, seems to be a mixture of those that can be compared at the same point in the continuous section and those that are not. However, since the tumor sample is very small, I understand that it is difficult to analyze. No more peer-reviewed rounds are needed.

Minor points.

1) The square part of the low-magnification photo of CD24 and the square part of the magnifying magnification are 90 degrees out of alignment. Please correct it as it is confusing.

Response: Sorry for the confusion. We have corrected the low-magnification photo of CD24 in the revised Figure 2. Please check!

2) The word Breast is missing in the graph on the upper right of Figure 4B.

Response: Sorry for the typo. We have corrected this in the revised Figure 4. Please check!

3) There is a misspelling in the upper left corner of Table 1. The term of "cancer" is "caner".

Response: Sorry for the typo. We have corrected "caner" into "cancer" in the revised Table 1. Please check!

4) The value of N-cadherin in Table 5 is 0.000. Please enter an appropriate value.

Response: To be more clear, we have changed P=0.000 into P<0.001. Please check the revised Table 5 for details.

**Reviewer #2:** Figure legends are non-existence or very brief. Keys of various symbols and color codes should be explained in the legends to allow a better appreciation of the results and hence the conclusions. The relationship between figures 1 and 2 needs to clearly stated. It will be helpful to indicate n values for various analyses when possible.

Response: As suggested, we have further provided more detailed information to the figure legends. Figure 1 shows the time intensity curve analysis of contrast-enhanced ultrasound (CEUS). The representative sections of CEUS were marked before surgery. Then, the tissue specimens were collected from these marked sections and subjected to immunohistochemical analysis of CSCs and EMT proteins (Figure 2). We have further clarified this in the revised manuscript. Please check!

Additionally, the n values have been indicated in the figure legends and table titles. Please check!
